# Functionalized Polymeric Nanoparticles for Yttrium Recovery by Chelating Effect

**DOI:** 10.3390/polym17152011

**Published:** 2025-07-23

**Authors:** Pedro Adrián Martínez-Montoya, Hugo Martínez-Gutiérrez, Ángel de Jesús Morales-Ramírez, Mónica Corea

**Affiliations:** 1 Instituto Politécnico Nacional, Escuela Superior de Ingeniería Química e Indústrias Extractivas, IPN-ESIQIE, Departamento de Ingeniería Metalúrgica, UPALM S/N Col. Lindavista, Gustavo A. Madero, Mexico City 07738, Mexico; adrianpedrom@gmail.com; 2 Instituto Politécnico Nacional, Escuela Superior de Ingeniería Química e Indústrias Extractivas, IPN-ESIQIE, Laboratorio de Investigación en Polímeros y Nanomateriales, UPALM S/N Col. Lindavista, Gustavo A. Madero, Mexico City 07738, Mexico; 3 Instituto Politécnico Nacional, Centro de Nanociencias y Micro-Nanotecnología, IPN-CNMN, UPALM S/N Col. Lindavista, Gustavo A. Madero, Mexico City 07738, Mexico; humartinez@ipn.mx; 4 Instituto Politécnico Nacional, Centro de Investigación e Innovación Tecnológica, CIITEC-IPN, Cda. de CECATI S/N, Santa Catarina, Azcapotzalco, Mexico City 02250, Mexico

**Keywords:** [Y] recovery, extraction process, stripping process, extraction selectivity, rare earths

## Abstract

Polymethyl methacrylate nanoparticles functionalized with three different compounds, acrylic acid (AA), curcumin (CUR), and fumaramide (FA), were tested in a two-step solid–liquid extraction process (extraction and stripping) for yttrium recovery. In both stages, the best conditions were determined: pH, solid–liquid ratio and the compound with the highest affinity for yttrium recovery, obtaining 90% of efficiency for both stages in a single work cycle. The results obtained by SEM ruled out the growing of nanoparticles by swelling and confirmed the formation of structural arrangements by the addition of the metal to the system. In addition, there is evidence that the recovery process can be selective considering the mixing of rare earth elements through changes in pH. Using isothermal titration calorimetry (ITC), the thermodynamic properties of the extraction process were calculated, understanding the system as the union of a macromolecule and a ligand. The results showed that the extraction process was spontaneous and highly entropic.

## 1. Introduction

The rare earth elements (REEs) are the most strategically important elements because they play a key role in many sophisticated technologies, such as the automotive industry, renewable energies, and defense sectors. They can contribute to increasing the efficiency and performance of products; however, it is well known that their future availability is uncertain [1]. Although their name suggests otherwise, rare earth elements are not scarce. They have high concentrations, and some elements exceed copper (Cu) in abundance. The REEs can be found in both primary and secondary mineral deposits [2].

Yttrium belongs to the REEs and more precisely to the group of heavy rare earth elements [3]. They are extensively used in a variety of applications, including televisions, fluorescent lamps, optical lenses, plasma display panels, and energy-efficient lighting (such as LEDs). They are also key components in superconductors, nuclear reactors, and electronic components for missile defense systems [2,4].

Since the year 2000, China has been main producer and exporter of rare earths, accounting for 90% of mining and refining. This is the case for yttrium, where 99% of world production comes from China. This has raised international concerns about supply risks, and it has been described as “critical” or “strategic” [5]. In order to address this, various sustainability strategies have been proposed, including urban mining and circular economy approaches. Urban mining involves processing urban solid waste to recover valuable materials, whereas the circular economy proposes a regenerative system that prioritizes the recovery and reuse of materials [6,7].

However, the future for rare earth elements is still bright, as exploration projects for new deposits have been promoted around the world since the last decade [4]. For example, in 2013, a large deep-sea mud deposit containing rare earth elements (including yttrium) was discovered in the northwestern Pacific Ocean near Japan. It has been reported that these resources have great potential for exploitation, and it is estimated that the amount of yttrium available could represent 62 years of the global annual demand and up to 780 years for the whole research area [8].

The extraction and treatment of rare earths generate environmental risks with an impact on human health. For example, when REEs are separated from the ore, a large amount of toxic chemicals, such as hydrochloric acid, ammonia, sulfates, etcetera, which produce wastewater, acid drainage, as well as large amounts of heavy metal residues are produced [9]. For example, owing to the chemical similarities among REEs, their separation is particularly difficult and requires highly selective and efficient techniques. One of the most employed methods for this purpose is liquid–liquid extraction, also known as solvent extraction. This technique is based on the transfer of REEs from an aqueous phase to an organic phase containing selective extractants, such as phosphoric acids (e.g., D_2_EHPA), carboxylic acids, amines, or organophosphines (such as Cyanex 272) [10]. Commonly used organic solvents include kerosene, toluene, or long-chain alcohols, which act as vehicles for the extractants and facilitate element-specific separation through multiple cycles of extraction, re-extraction, and stripping, where high-purity rare earths can be obtained. The effectiveness of this process directly impacts the overall efficiency, cost, and environmental footprint of REE production. Protein-based processes have even been proposed for the recovery and separation of rare earth elements. Other techniques, such as ion exchange and membrane separation, have proven to be less efficient for the accurate separation of REEs. Ion exchange is slow, requires expensive resins, and has limited loading capacity. Membrane separation, on the other hand, has low selectivity and poor chemical resistance of membranes to highly acidic solutions used in rare earth treatment [11,12]. Both ion exchange and membrane technologies tend to be less environmentally sustainable, as they can generate waste that is difficult to handle or requires high energy consumption. Currently, the development of work involving polymer-based extraction materials for REE extraction is increasing. These methods are a more sustainable alternative to current industrial methods, including solvent-impregnated resins (SIR) [13], microcapsules, polymeric inclusion membranes (PIMs), polymeric inclusion microspheres (μPIBs), nanomaterials, and nanoparticles based on polymers. This extraction materials offer the advantage of more economical use compared with the extractants used in industry [14,15,16]

A more sustainable and environmentally friendly alternative to traditional rare earth mining is recycling, also known as urban mining. This approach involves the recovery of rare earth elements (REEs) from discarded electronics and other technological waste [17,18,19,20]. Unlike conventional mining, which involves the extraction of large volumes of ore with considerable environmental impact, urban mining presents itself as a more efficient option with a smaller environmental footprint. Urban mining is related to cleaner and more selective extraction mechanisms, hydrometallurgical methods with less toxic reagents, and even emerging technologies, such as bioleaching [12].

Compared with traditional mining, these techniques not only significantly decrease the generation of solid waste and toxic emissions, but also promote a circular economy where resources are reused and the life cycle of materials is extended. Therefore, recycling REEs through urban mining represents not only a viable technical solution, but also a fundamental step toward a more sustainable and responsible industry. For example, neodymium–iron–boron (NdFeB) magnets are present in hard disks, electric motors, and wind generators. These contain up to 30 wt.% of rare earths. Fluorescent lamps and energy-saving light bulbs contain lanthanum, europium, and terbium in phosphors. Nickel–metal hydride (NiMH) batteries used in hybrid vehicles contain lanthanum and other REEs. Waste electronic equipment (WEEE), such as cell phones, tablets, and laptops, can contain up to 0.2 g of REEs per unit, especially in components such as screens, speakers, and micro components [21].

This work proposes an innovative mechanism for the recovery of yttrium using functionalized polymeric nanoparticles, evaluated in model solutions. The proposal is based on the synthesis of polymethylmethacrylate (PMMA) nanoparticles functionalized with acrylic acid (AA), curcumin (CUR), and fumaramide (FA) on their surface, where the functional groups of each compound act as chelating sites capable of coordinating with metal ions, forming stable metal complexes and allowing for the recovery of the metal.

This approach represents a potential alternative to traditional rare earth recovery methods, which are often polluting and inefficient. Since rare earth elements exhibit a high affinity for oxygen (oxyphilicity), they can easily form coordination bonds with ligands containing oxygen atoms. Carboxyl groups present in acrylic acid have been reported [21] to confer to nanoparticles a high affinity for rare earth ions. Moreover, previous studies have shown that these carboxylic ligands are effective for both yttrium [Y^3+^] and europium [Eu^3+^], functioning as excellent adsorbents in selective recovery processes [21].

## 2. Materials and Methods

### 2.1. Materials

The used functionalized nanoparticles were obtained by means of emulsion polymerization techniques. Table 1 shows the used materials. The detailed polymerization methodology was reported in a previous work [21]. Three series of latex with three different particle contents were tested (5, 10, and 15 wt.%) and three functional groups acrylic acid (AA), curcumin (CUR), and fumaramide (FA) (at 5 wt.% concentration with respect to the total quantity of monomer) were put on the surface of particles.

A synthetic yttrium solution was prepared from YCl_3_·6H_2_O, (Yttrium (III) chloride hexahydrate), purchased by Alfa Aesar, Reaction^®^, 99.99% (REO, Haverhill, MA, USA), at 1000 ppm of yttrium [Y] in aqueous media.

### 2.2. Characterization Techniques

#### 2.2.1. Dynamic Light Scattering and Zeta Potential

The particle size distribution was measured by dynamic light scattering (DLS) at 90°, and by electrophoresis, the zeta potential (ζ) and the latex stability were measured. Both measurements were conducted using a Zetasizer Nano ZSP equipment from Malvern Instruments (Malvern, Worcestershire, UK). For that, 0.084 g of latex was diluted in 10 mL of deionized water. The solution was titrated with a YCl_3_ solution at 15 mM using a Multi-Purpose Titrator (MPT-2 of Malvern Instruments, Malvern, Worcestershire, UK). For each titration, the changes in the average particle size, zeta potential, and pH were determined. The experiments were performed in quadruplicate at 25 °C.

#### 2.2.2. Scanning Electron Microscopy (SEM)

The morphology of the polymeric nanoparticles was analyzed after the extraction process by scanning electron microscopy (JEOL JSM-7800F, Joel Company, Tokyo, Japan). The latex was diluted and dried onto the base of a cylindrical copper specimen holder for 1 h. After that, the polymeric particles were covered with a thin layer of gold for at least 15 s. The sample was analyzed at different magnifications (5000, 20,000, and 50,000) at 5 kV, WD 6 mm, UED, with a 30–40 s gold coating [21].

#### 2.2.3. Isothermal Titration Calorimetry (ITC)

The calorimetric thermodynamic properties of the interaction between polymeric nanoparticles and metal (yttrium) were measured by ITC. The experiments were performed in an Automated MicroCal PEAQ-ITC from Malvern Panalytical (Boston, MA, USA). A 200 μL cell was filled with the nanoparticle dispersion at 1 mM with respect to functional group and a 40 μL titration syringe was filled with yttrium solution at 15 mM. The samples were degassed with nitrogen. The used parameters for each experiment were as follows: 39 injections, cell temperature of 25 °C, stirring speed of 750 rpm, volume of each injection of 1 μL, and duration of each injection of 10 s.

### 2.3. Extraction Process and Stripping of Yttrium

A mixture consisting of 1 g of latex and 10 mL of an aqueous solution containing 1000 mg/L of yttrium (solid-to-liquid ratio of 0.1 g/mL) was subjected to constant stirring at 400 rpm for 2 h. Upon completion of the extraction process, the polymeric particles (now loaded with metal ions) were separated via centrifugation. It is important to note that the functional groups present in the particles were not used in combination. The stripping process was performed using distilled water adjusted to pH 2, 4, and 6 modified with drops of concentrated hydrochloric acid (HCl), and pH 8 modified with drops of concentrated ammonium hydroxide (NH_4_OH). The used latex–aqueous ratio for the stripping was the same as that in the extraction process. Finally, a kinetic study was carried out for the extraction and stripping processes at room temperature. The yttrium content of both processes was analyzed by inductively coupled plasma–mass spectrometry (ICP-MS), Perkin Elmer (Waltham, MA, USA). The extraction percentage (%E) and stripping percentage (%S) are defined as follows:

(1) $$\%E=\frac{\left[M_{i}\right]-[M_{e}]}{\left[M_{i}\right]}\times 100$$

(2) $$\%S=\frac{\left[M_{e}\right]-[M_{s}]}{\left[M_{e}\right]}\times 100$$

where $\left[M_{i}\right]$ represents the metal cation initial concentration in aqueous phases (1000 mg/L), ${[M}_{e}]$ is the metal cation concentration in aqueous phases after equilibrium, and $\left[M_{s}\right]$ is the aqueous concentration of metal cations after stripping.

## 3. Results and Discussion

Three series of latex were obtained by means of emulsion polymerization techniques (where the latex is defined as a colloidal dispersion of polymeric particles in aqueous media [22]). The contents of latex particles were 5, 10, and 15 wt.%, where a functional group was put on the particle surface (acrylic acid, fumaramide, or curcumin). The obtained particles presented a spherical shape with an average size close to 100 nm and they were monodispersed. The physicochemical and morphological characterization of the used functionalized polymeric particles in the yttrium recovery process were reported in a previous work [21].

### 3.1. [Y] Extraction Process

**Figure 1:**
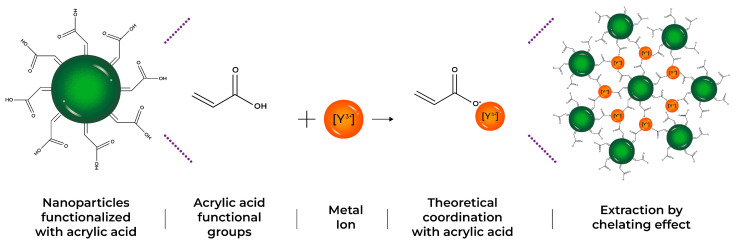

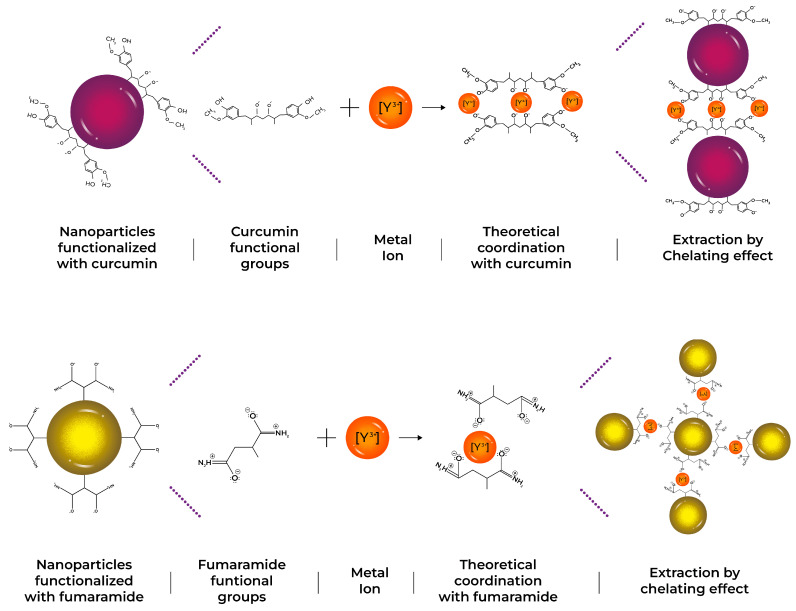
Theoretical scheme of yttrium extraction, by the formation of chelating complexes for three different systems: AA, CUR, and FA.

All synthesized latexes were tested in the [Y] extraction process. A latex ratio (1 g) with an aqueous volume of (10 mL) at a concentration of 1000 mg/L of yttrium was mixed (solid/liquid ratio 0.1 g/mL). Figure 1 shows a scheme of the interaction between functional groups of nanoparticles and metal ions during the extraction process, helping to better understand the formation of polymeric particle–metal systems.

**Figure 2:**
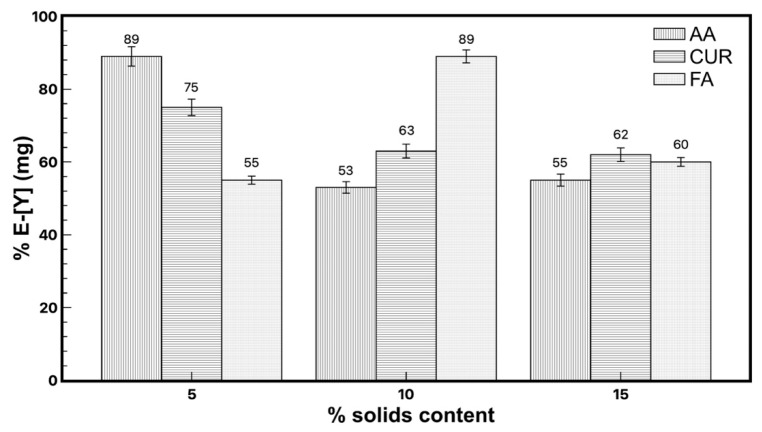
Yttrium extraction with the three different latex series, modifying the solids content.

The initial pH values of each emulsion containing the functionalized particles were 3.9, 3.8, and 2.9 for AA, FA, and CUR, respectively, which were obtained directly from the synthesis. The experiment to determine the recovery efficiency of particles was conducted taking an aliquot from the aqueous supernatant of the mixture between the latex and metal solution and was measured by ICP to determine the quantity of yttrium remaining in the water. The extraction results are shown in Figure 2. It is observed that the recovery efficiency of yttrium reached values from 50% to 89%.

**Figure 3:**
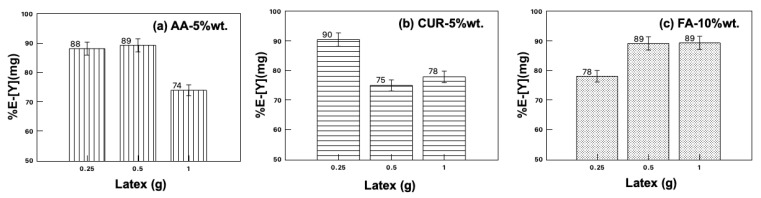
Yttrium extraction with the three different latex series, modifying the solid/liquid ratio.

The maximum extraction of yttrium (89%) was observed for the functionalized nanoparticles with acrylic acid and fumaramide close to 10 wt.%, while that of functionalized curcumin reached 75% extraction with a 5 wt.% particle content. Figure 3 shows the results of the recovery efficiency of all latexes with a 5 wt.% particles content as an example, and where the solid/liquid ratio was modified between 0.25 and 1. It was expected that increasing the number of nanoparticles in the system would also increase the extraction percentage, but this only happened in the case of fumaramide, where the extraction slightly increases as the nanoparticle content increases. In the case of AA-5wt.% and CUR-5wt.%, the extraction percentage of yttrium decreased as the number of nanoparticles increased in the system.

This behavior can be explained by agglomerate formation by the interaction with the metal ions, blocking the active sites and inhibiting the contact of the functional groups with the metal ions, resulting in lower extraction percentages.

The good extraction efficiency of [Y] obtained with the acrylic acid and fumaramide as functional groups in the nanoparticles is mainly attributed to two parameters that influence the effectiveness of the technique. The first is the affinity of metal to the functional groups of acrylic acid and fumaramide. This allows for the coordination of the metal ion quickly by means of ion–dipole phenomena. The second parameter is the pH of the medium. This last one has an important influence on the efficiency of the extraction systems and can alter the acid/base equilibria of the metallic species and the extractants; consequently, it will affect their ability to form complexes [23]. According to the species distribution diagram, the presence of the [Y^3+^] species is favored when the medium is acidic (pH > 4) [13].

This cation is attracted by the carboxylic groups of the acrylic acid and the amide groups from the fumaramide, which are located on the surface of the nanoparticles. These functional groups begin to ionize in the presence of metal ions, forming a dipole. When the pH increases, the carboxylic groups are also ionized, which allows electrostatic interactions to form a non-covalent complex called a chelate with the metal [24]. In a general way, the functionalized nanoparticles act as the chelating extractants. As hydrogen ion donors, the chelating extractants recover metals by an ionic exchange mechanism similar to the one presented in Equation (3) and represented by Figure 1, which exemplifies the process of formation of the chelating complexes and the interaction of the functional groups with the metal ions. But the resulting organic complexes are stabilized by the organic anion coordinating the central cation in at least two positions [25,26,27].

Equation (3) describes the general extraction of rare earth elements from aqueous media by ionic exchange in their acid form, and is generally expressed as:

(3) $${Ln}^{3+}+3\overset{̿}{HA}=\overset{̿}{LnA_{3}}+3H^{+}$$

where Ln represents any rare earth, A denotes the organic anion, and the double line represents species present obtained in the organic phase [25]. The obtained high percentage of yttrium extraction is attributed to the efficiency in the formation of chelating complexes. The results showed that the carboxyl and amide groups located on surfaces of particles are the best candidates for yttrium separation and purification, since yttrium can be separated from the lanthanide extraction series in systems using carboxylic acids [11,25]. It has been reported from FTIR results that coordination between the carboxylate group (COO-) and metal ions occurs during the extraction of rare earth elements [21,28]. This increases the effectiveness of the extraction and with 2.5 < pH < 3.5; therefore, this work will focus only on the latexes that achieved the highest recovery rates, AA-5%wt., FA-10%wt., and CUR-5%wt. (percentage of solids content), under the same conditions.

### 3.2. UV-Vis

**Figure 4:**
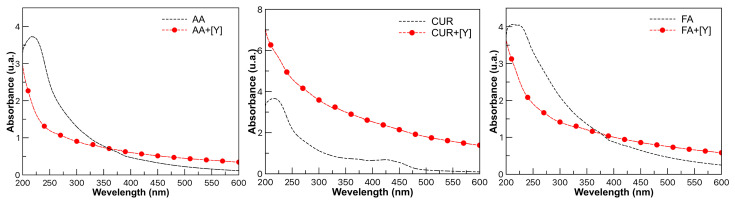
UV-Vis spectra of latex with AA, FA, and CUR (black dotted line) all at 10 wt.%. Spectra of latex after extraction process (red line).

In order to know the effects of the coordination mechanism of yttrium with the functional groups, a UV-Vis analysis was performed before and after extraction. In Figure 4 (black dotted line), bands in the 200–600 nm range attributed to carboxyl groups, amides, esters, and aromatic compounds, corresponding to the three latex series AA, FA, and CUR, respectively, are observed. The description of each functional group has been reported in detail in a previous work [21].

In Figure 4, red lines show the bands of the same compounds after the extraction process with yttrium. The formation of the coordination complexes between the metal and each functional group is reflected in the decrease or increase in the maximum absorbance. In the case of AA, a maximum absorbance of 222 nm was observed, attributed to the carboxylic groups as an auxochrome (COOH) group, which is displaced and no longer observed after the extraction process. For CUR, a shift at 430 and 480 nm was observed, which was attributed to the aromatic and phenolic groups peculiar to curcumin, which are no longer observed after interaction with yttrium. The maximum peak at 218 nm is shifted and can no longer be observed. The FA spectrum presented an absorbance maximum at 230 nm attributed to the presence of the amide group (-CONH_2_), which, after the extraction process, shifts and is no longer observable [29,30].

### 3.3. Dynamic Light Scattering and Zeta Potential

**Figure 5:**
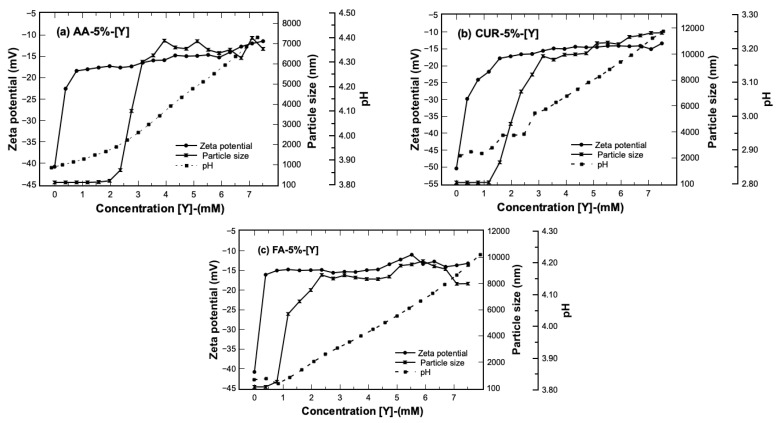
Average particle size, Z–potential, and pH as a function of the concentration of [Y] added to the system. Solid/liquid ratio = 0.1 g/mL, [Y] concentration = 0.015 mol/L.

The behavior of the latex–metal complex was analyzed during the extraction process by means of dynamic light scattering and zeta potential. The experiment was carried out keeping the ratio of nanoparticles constant in the system and injections of metal solution were added at a concentration of 15 mM. In this way, measurements of changes in size, stability, and pH as a function of the total yttrium content on the solution were performed. The results of three parameters of average nanoparticle size, Z potential (complex stability), and pH of the three functional groups as a function of concentration of yttrium are presented in Figure 5.

The results show that the average nanoparticle size before the extraction process had a diameter close to 100 nm and a polydispersity index close to 1. This means that the particle system does not present agglomeration [21]. However, the nanoparticle diameter began to grow as [Y] was added to the system, reaching sizes between 7000 nm and 10,000 nm. The increment in size could be attributed to a possible agglomeration of the nanoparticles of them during the extraction process, or maybe to the formation of structural arrays by the interaction with yttrium ions.

Another parameter to confirm the behavior of the systems is the measure of zeta potential, which increases its value with the increase in yttrium concentration. The latex presented initial values of −40 < *ζ*/mV < −50, which means that, at the beginning of the extraction process, the nanoparticles are colloidally stable by the electrostatic repulsion forces between them, steric hindrance, or a possible combination of both phenomena [31].

The stability of the latex is broken when the extraction process begins and as the metal content in the medium increases. This means that the particles–metal interaction becomes stronger, and the stability is apparently lost, reaching a final value of −10 mV for all three latexes. This change could be associated with the growth of the nanoparticles, and they begin to precipitate at the end of the extraction process. The behavior of the nanoparticles with other rare earth ions has been reported with similar results, attributing the increase in the Z-potential of the system to the formation of metal complexes during the extraction step [21].

The change in pH of the system during the extraction stage is another variable that was measured, and the results are presented in Figure 5. The latex–metal system starts with a pH value of 3.9, 3.8, and 2.9 for AA, FA, and CUR, respectively, directly obtained from the synthesis. At the end of the extraction process, the reached values were 4.4, 4.2, and 3.2 for the same materials. This behavior is attributed to the chelating complexes’ formation (functional groups and metal ions). For example, H^+^ ions are released into the aqueous solution while the metal cations form a chelate with the carboxylic groups, affecting the pH value of the solution. As mentioned above, throughout the extraction process, it is desirable to maintain a range of acid pH (2–6), which favors the extraction of metal ions.

Finally, there is a relationship between the pH and Z-potential, and it is associated with the value of pKa of the functional groups. The degree of ionization in a polymer with weakly ionizable functional groups changes at a specific pH known as pKa [32]. When the pH of the medium is modified $\left({pH}_{\frac{1}{2}}\right)$, functional groups can accept or donate H^+^ protons (as occurs with AA or CUR), changing the degree of ionization and the net charge of the polymer chain [32,33]. It has been reported that pH-sensitive groups have a pKa value close to 4.5 [34]. In this case, the $\left({pH}_{\frac{1}{2}}\right)$ values for all three latex systems are less than the pKa value so the functional groups are predominantly uncharged (electrically neutral molecules). All three latex series tended to increase the Z potential value (as observed in Figure 5) as the metal concentration in the system increased. This indicates that the functional groups interact with the yttrium ions, reducing the electrostatic repulsions and decreasing the stability of the system.

### 3.4. ${pH}_{\frac{1}{2}}$ and Extraction Selectivity

In metal-extraction processes with organic extractants, the pH value in the middle of the extraction process is defined as $\left({pH}_{\frac{1}{2}}\right)$ and is closely related to the extraction capacity of the extractant [11,35]. This value was also obtained in the extraction process with the functionalized nanoparticles a, where a ${pH}_{\frac{1}{2}}$ value of 4.2 is reached for yttrium. Therefore, the pH change that occurs in the system is favorable if it remains in the range where the extraction is efficient. As can be seen in the species distribution diagram [13], the pH value is within the range where [Y^3+^] species is found, giving a wide range for extraction.

For example, a higher ${pH}_{\frac{1}{2}}$ value indicates a weaker extraction capacity. Values of ${pH}_{\frac{1}{2}}$ for different rare earth elements have been reported with different extractants [11], coinciding with the results obtained in this work, where a pH range between 3.8 and 4.4 was found.

**Figure 6:**
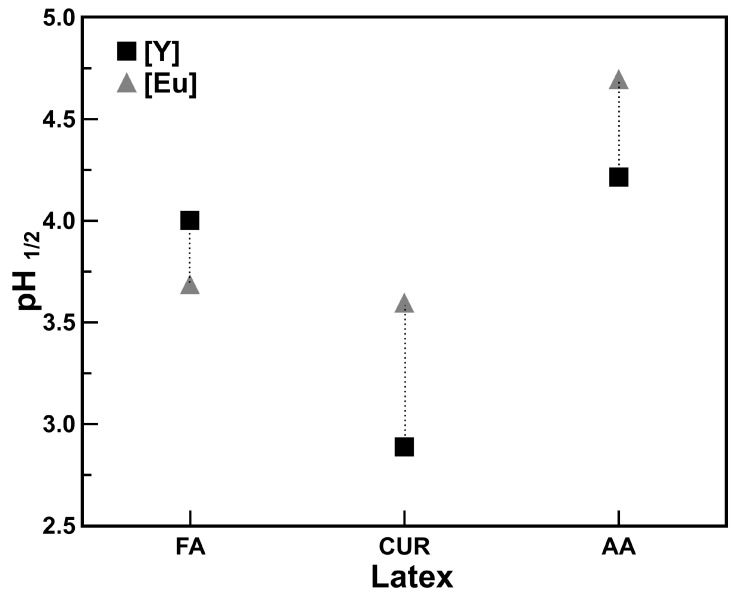
${pH}_{\frac{1}{2}}$ ranges for selective yttrium and europium extraction. with each latex series. (solid/liquid ratio 0.1 g/mL).

If the ${pH}_{\frac{1}{2}}$ extraction value for [Y^3+^] is different from that for the other rare earth elements, for example Eu, and where the functionalized polymeric nanoparticles had higher affinity for yttrium extraction over the rest of the lanthanides, this means that the element can be separated from the rest of Ln^3+^, as is the case for the Eu in combined solutions, considering a batch extraction process. This is shown in Figure 6, where the ${pH}_{\frac{1}{2}}$ values for yttrium and europium are compared with each latex series, and two different ranges can be observed. The latex series that presents the greatest difference between ${pH}_{\frac{1}{2}}$ values is the one with curcumin, which is, at the same time, the one that presents the highest yttrium extraction. This wide difference in ${pH}_{\frac{1}{2}}$ values between yttrium and europium allows for the consideration of the extraction by combining both metals in the same solution, and controlling the pH value can make the extraction selective.

#### Extraction Selectivity [Y] vs. [Eu]

**Figure 7:**
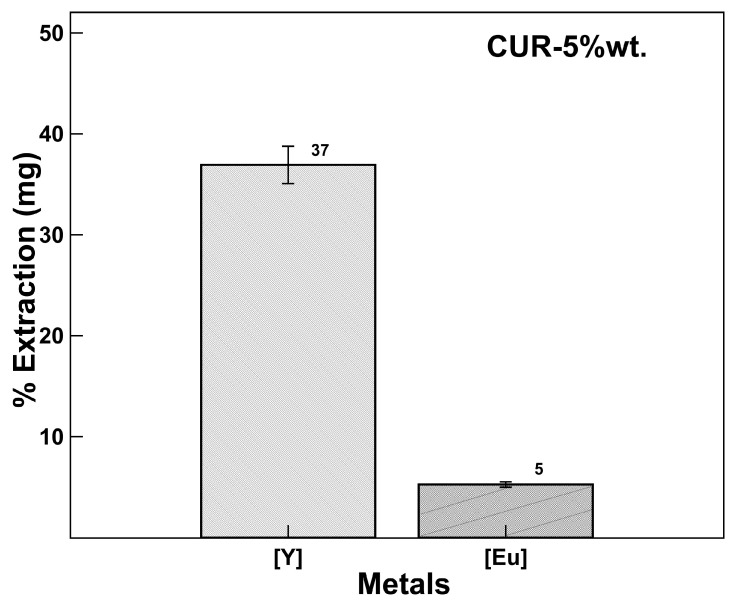
Extraction selectivity [Y] vs. [Eu]. CUR-5wt.%, concentration of 1000 ppm (solid/liquid ratio 0.1 g/mL).

A selectivity experiment was performed controlling the ${pH}_{\frac{1}{2}}$ at 2.8, values reported for an efficient yttrium extraction process. Figure 7 shows the yttrium recovery percentages with respect to europium in the same mixture at the same concentration, obtaining an yttrium extraction percentage of 37% with a europium co-extraction percentage of 5%, much lower than reported with ionic liquids [36,37].

This result demonstrates that it is possible to establish a pH-dependent selective extraction mechanism coinciding with that reported with extractants such as Cyanex 572 (commercial sample supplied by Cytec Inc.) [36] used for the extraction of rare earths and with higher separation efficiency.

The percentage of yttrium recovery obtained in the mixed extraction is attributed to the competitiveness that exists between the metal ions to occupy the active sites in the nanoparticles, favoring the yttrium. The experiments were performed in triplicate, and it was difficult to control and adjust the pH value throughout the process, so the percentage of yttrium recovery seemed to be low, although the process was designed in cycles of work; two cycles would be sufficient to extract more than 70%, so this is left for future work.

To analyze the growth of the nanoparticles observed via DLS, the obtained systems were observed by SEM, confirming the stability results of the materials.

**Figure 8:**
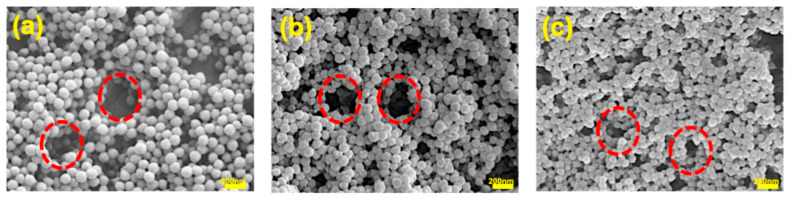
SEM images at 50,000× of functionalized polymeric nanoparticles with (**a**) AA-5wt.%, (**b**) CUR-5wt.%, and (**c**) FA-15wt.% after the extraction process with [Y]. Arrangements as pearl necklaces are also observed in the polymeric particles (indicated for red dotted circles).

The micrographs are shown in Figure 8, observing that the nanoparticles had a spherical morphology, homogeneity, and without agglomeration. Arrangements as pearl necklaces are also observed in the polymeric particles (indicated for red dotted circles), which is reflected as an increment in the average size by dynamic light-scattering and a destabilization of the latex–metal system with an increment in the value of the Z potential.

This behavior is attributed to the yttrium metallic atom being bound to the coordination atoms (or donor atoms) of the ligands (carboxyl, amide, and hydroxyl). These ligands coordinate the metal atom with more than one atom to form a ring [38]. This ligand is known as a chelating ligand and the formed metal complexes are metal chelates [21,39]. Therefore, the nanoparticles do not increase in size, but form associations by the interactions between the functional groups located on the surfaces of the nanoparticles and the yttrium metal ions. 

### 3.5. [Y] Stripping Process

**Figure 9:**
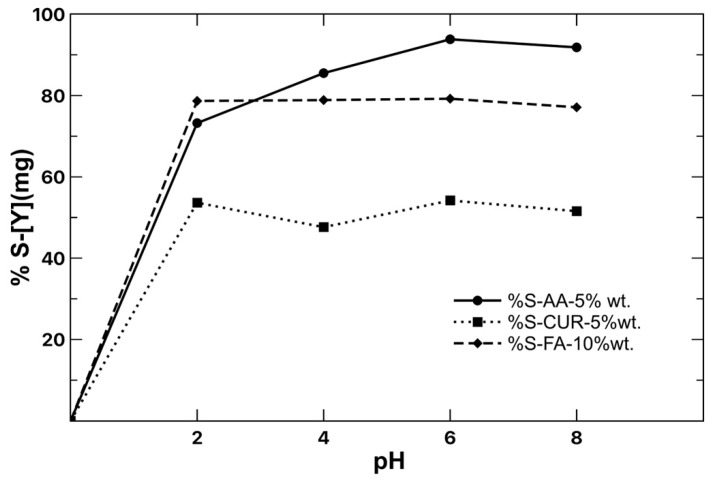
Stripping of [Y] with varying pH, 120 min, and 1 g latex per 10 mL of water. [Y] 900 mg/L initial concentration.

Stripping is understood as an inverse process to extraction, where the aim is to release the chelated metal ions to the medium. This leaves free active sites on the surface of the nanoparticle for reuse. To achieve this, the pH is the most important parameter in the process, which helps to release the yttrium ions from the polymeric particle. Therefore, values ranging from 2 to 8 were tested. Figure 9 shows the pH change of the medium for the stripping process. The results show that the three latex series presented higher stripping at pH of 6, with AA being the one with the highest efficiency (90%) for yttrium in a single cycle. The stability of the formed complex during the extraction process was weakened for the modification to pH, so it began to decompose, releasing [Y^3+^], again leaving active sites on the surface of the nanoparticle for a new extraction process.

During the stripping process, the functional groups of the compounds are protonated again, taking H^+^ from the aqueous medium. In this way, there is an exchange by electrostatic interactions that results in the release of the metal ions. This behavior is observed in the three different series of nanoparticles, with the difference that the process takes place with greater or lesser speed, depending on the structure of each compound; therefore, AA, having a simpler molecule, releases the metal faster compared with CUR, which, having two aromatic rings in its structure, does release the metal, but more slowly.

### 3.6. Isothermal Titration Calorimetry (ITC)

Isothermal titration calorimetry (ITC) has been used for the characterization of binding phenomena at equilibrium [40]. ITC involves the detection of produced (exothermic) or absorbed (endothermic) heat, when binding or interaction occurs between two molecules: in this case, between functionalized groups in the nanoparticles and yttrium metal ions. The ITC data were collected using the accompanying software and corrected for the heat of dilution by subtracting the heat remaining after the saturation of the binding sites.

The analysis of the data was performed by a complete mathematical solution to the titration method, which includes the description of the binding reactions of a ligand and a macromolecule. The complete development of the utilized model has already been reported, as well as the fit to the titration isotherm [40,41], and has been proposed as a binding model to describe the chelation of high-affinity metal ions [42].

Isothermal titration calorimetry (ITC) data allow for the determination of several thermodynamic parameters, including the reaction stoichiometry (N), which represents the number of metal ions bound per unit of adsorbent, and the equilibrium binding constant ($k_{D}$). Additionally, the enthalpy change (ΔH) can be calculated, indicating the amount of heat released or absorbed during the binding process. ITC also enables the determination of entropy (ΔS), which reflects changes in the overall disorder of the system, and the Gibbs free energy change (ΔG) of the process, calculated using Equation (4) [43].

(4) $$\Delta G=-RT\ln k_{D}=\Delta H-T\Delta S$$

**Figure 10:**
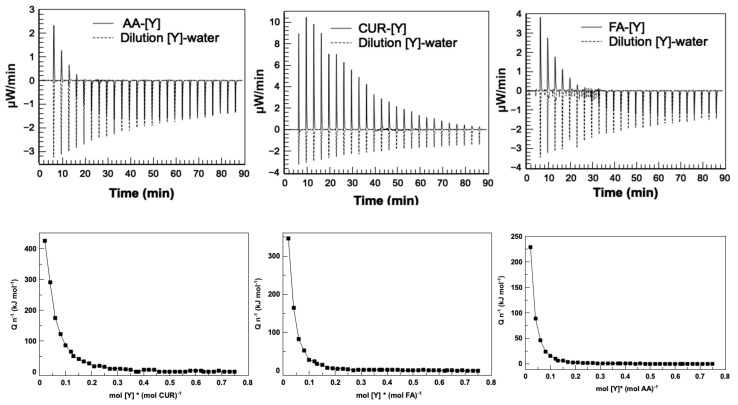
Thermograms (**top**) and binding isotherms (**bottom**) for the determination of thermodynamic parameters of the latex–metal bond. Heat molar ($Qn^{-1}$) as a function of molar ratio between metal ions and functional groups.

The thermograms of the latex–metal complex interactions and the metal–water interaction for the three latex series are shown in Figure 10. The results show that the AA-[Y] and FA-[Y] complexes had similar behavior, showing strong endothermic peaks at the beginning, attributed to the ion formation in the functional groups and the attraction of metal ions; followed by exothermic effects that decrease until reaching “the system saturation”. This is interpreted as the occupancy of yttrium in all active sites of the nanoparticles, suggesting that the adsorption of metal ions to the nanoparticles sites is very quick.

The CUR-[Y] complex showed endothermic adsorption signals with decreasing intensities until saturation of the system was reached. The endothermic nature of the signals could first suggest resonance effects in the aromatic rings of curcumin, when the metal ion is recognized by its hydroxyl and ether groups, which causes the formation of negative charges of these groups and the coordination of the metal forming the complex. It can be corroborated because it has been reported that chemical contributions (interactions of metal ions with functional groups, intermolecular hydrogen bonds, electrostatic interactions, or other chemical reactions, such as precipitation) lead to an exothermic effect, while physical contributions (dehydration effects, structural rearrangement, or electrostatic effects) lead to endothermic effects as in metal adsorption systems [44].

The obtained molar ratio from the calorimetric calculations allows for obtaining the maximum absorption capacity for each compound. Table 2 shows the necessary concentrations of each compound to extract the same amount of metal.

From the thermograms, the thermodynamic properties of the latex–metal systems were calculated by means of nonlinear least squares [45]. The entropy (ΔS), enthalpy (ΔH), and Gibbs free energy (ΔG) of the three interaction system extractions fit and they are shown in Table 3. A positive enthalpy value is characteristic of the strong endothermic effect associated with the adsorption of the metal with the functional groups [44], which suggests that the three series of functionalized nanoparticles can form complexes with [Y^3+^]. A positive entropy value is considered favorable for metal–ligand interaction processes [46], which suggests in all three cases that the binding reaction is entropy-driven.

The thermodynamic results indicate that the extraction system with acrylic acid presents the highest feasibility, reflected in the lowest values of ΔG and ΔH. This efficiency is attributed to the electrostatic interactions between the functional groups of the acrylic acid and yttrium ion. Meanwhile, curcumin- and fumaramide-based systems show higher values of ΔG and ΔS, because the complexes formed with yttrium are mainly stabilized by ion–dipole induction, which is a type of intermolecular interaction that occurs when a positive ion (metal ion) approaches an electrically neutral but polarizable molecule (nanoparticle), causing a distortion in the electronic cloud of the particle. This distortion generates an induced dipole, which interacts electrostatically with the yttrium metal ion. Therefore, the entropy driving the process is the pure interaction between the functional groups in the nanoparticles on the surface and metal ions [47,48].

Evidently, differences in the interaction mechanisms exist, but the three systems exhibit similar positive entropic contributions, which is related to the increase in molecular mobility and the appearance of net charges on the functional groups when forming metal complexes by the chelating effect.

The association constant $\left(k_{D}\right)$ is the extreme affinity range [49], so the calorimetric results prove the efficient extraction, attributed to the strong interactions between the functional groups of the compounds AA, CUR, FA, and the [Y^3+^] ions.

Finally, the stoichiometry of the reaction, or the stoichiometry parameter N, is similar between the AA-[Y] and FA-[Y] complexes, and different for the CUR-[Y] complex, as can be seen in Table 3. The stoichiometry parameter N has been interpreted in two main ways. The first one, as the generalization, is that the stoichiometry N represents the titrand with N equivalent and independent binding sites for the titrant. The second one is that the stoichiometry N accounts for the possibility of having a percentage of the titrant in a non-competent binding conformation with the titrant [46].

The first interpretation tells us that the number of [Y^3+^] bonds with each AA functional group is 0.32; in other words, the stoichiometry is the reciprocal, which would be 3 (three hydroxyl groups for one [Y]). This is given by deprotonation of the hydroxyl group to form stable complexes.

In the case of the complex with fumaramide, this is because the delocalization of the electrons in the amide groups negatively charges the oxygens of enolic groups with a stoichiometry of 3, similar to the complex formed with AA.

## 4. Conclusions

Functionalized nanoparticles have been tested using the two-step solid–liquid extraction technique (extraction and stripping) for yttrium recovery. The best conditions have been determined for both stages: A pH range between 2 and 3 is ideal for the extraction process with acrylic acid-functionalized nanoparticles and a solid–liquid ratio of 0.1 mg/mL, reaching recovery percentages of 90% (900 mg/L-[Y]), attributing this behavior to the chelating effect. It was determined that the stripping stage at pH 6 has a higher release of metal ions, with a percentage of 90% in the case of AA in a single work cycle (810 mg/L-[Y]).

To understand the extraction mechanism, titrations were performed by measuring pH, Z-potential, and particle size constantly throughout the process. This was further confirmed by SEM images. The formation of these structural arrangements results in the destabilization of the system from a value of −40 < *ζ*/mV < −50 to a value of −10 *ζ*/mV. By means of DLS, sizes up to 10,000 nm are obtained, which causes the precipitation of the nanoparticles when they are saturated with metal. Continuous pH analysis during the extraction process has allowed for determining a pH range from 3.8 to 4.4 for yttrium extraction, while for europium, it is 4.8 to 5.3. This makes the process selective with a real mixture of these metals in mind. Finally, by means of ITC, it has been possible to calculate the thermodynamic variables of the extraction system. This technique has made it possible to determine the stoichiometry of the reaction N interpreted as the molar ratio between functional group and metal of 0.41, 1.17, and 0.40 for AA-[Y], CUR-[Y], and FA-[Y], respectively, the equilibrium bond association constant $k_{b}$, and the enthalpy change (ΔH). In addition, it has been possible to determine the entropy (ΔS) and the Gibbs free energy of the process (ΔG); this shows that the extraction processes of all three systems, AA-[Y], CUR-[Y], and FA-[Y], are highly entropic and spontaneous.

## Figures and Tables

**Table 1:** Materials used and the polymerization methodology for latex preparation.

Reagents and Purity	Source	Reagent Use	^b^ Latex Preparation
Methyl methacrylate (MMA)	Especialidades Químicas, S.A. de C.V., MEXICO	Monomer	^c^ First stage: a pre-emulsion containing MMA, initiator, surfactant, and crosslinking agent was added with a dosing pump at a flow rate of 0.3 g/min, at 75 °C, controlled by a thermal bath and nitrogen atmosphere.
Nonylphenol ethoxylate ammonium sulfate	Abex^®^ EP 120, Solvay from USA	Surfactant	
Potassium persulfate	Sigma-Aldrich from USA	Initiator	
Ethylene glycol dimethacrylate (EDGMA)		Crosslinking agent	
^a^ Acrylic acid (AA) ≥ 99%		Functionalization	^c^ Second stage: the functional group was added to ensure that it would remain on the surface of particle.
^a^ Curcumin (CUR) ≥ 65%			
^a^ Fumaramide (FA) ≥ 96%	ChemCruz from USA		

^a^ Were used as received. ^b^ Was prepared by means of emulsion polymerization in a semi-continuous process in two stages. ^c^ The dispersion medium was deionized water.

**Table 2:** Necessary concentration of functional group to reach saturation at the same concentration of yttrium.

System Extraction	Functional Group Concentration (mol)	Yttrium Concentration (mol)
AA-[Y]	0.5	0.015
CUR-[Y]	0.46	
FA-[Y]	0.56	

**Table 3:** Thermodynamic parameters of latex–metal macromolecule formation as determined by isothermal titration calorimetry at 25 °C.

System Extraction	$K_{D}(M\;{\times \;10}^{-6})$	N	$\Delta G(\frac{kJ}{mol})$	$\Delta H(\frac{kJ}{mol})$	$-T\Delta S(\frac{kJ}{mol∙K})$
AA-[Y]	42.2	0.32	−25	2.68	−27.7
CUR-[Y]	253	0.97	−20.5	8.92	−29.5
FA-[Y]	134	0.33	−22.1	6.76	−28.9

## Data Availability

https://docs.google.com/spreadsheets/d/1D7KL7wIOnJ1t05SeR7_KHH8lCB0tf9i8/edit?usp=sharing&ouid=115454450837313585789&rtpof=true&sd=true (accessed on 9 June 2025).

## Abbreviations

The following abbreviations are used in this manuscript:

AA	Acrylic acid
CUR	Curcumin
FA	Fumaramide
REEs	Rare earth elements
